# Anti-N-methyl-D-aspartate receptor encephalitis presenting as atypical psychosis in multiple sclerosis: a case report

**DOI:** 10.1186/s12888-021-03351-7

**Published:** 2021-07-12

**Authors:** Khushminder Chahal, Tara Endeman, Sarah Scapinello, Michal Sapieha

**Affiliations:** 1 Guelph General Hospital, 115 Delhi St, Guelph, ON N1E 4J4 Canada; 2 Homewood Health Centre, 150 Delhi St, Guelph, ON N1E 6K9 Canada; 3 Department of Psychiatry and Behavioural Neurosciences, McMaster University, 100 West 5th St, Hamilton, ON L8N 3K7 Canada; 4 Department of Psychiatry, University of Ottawa, 5457-1145 Carling Ave, Ottawa, ON K1Z 7K4 Canada

**Keywords:** Psychosis, Autoimmune encephalitis, NMDAR encephalitis, Multiple sclerosis, Demyelinating syndromes, Immunotherapy

## Abstract

**Background:**

Anti-N-methyl-D-aspartate receptor (anti-NMDAR) encephalitis is an autoimmune disorder which often presents with neuropsychiatric symptoms. A large proportion of cases are associated with an identifiable tumor, most commonly ovarian teratoma. However, recent literature has also described an overlap of anti-NMDAR encephalitis and demyelinating syndromes. Cases have been reported of anti-NMDAR encephalitis in patients with ADEM, optic neuritis, myelitis and multiple sclerosis. This link is considered rare, however has important clinical implications as treatments and prognosis may differ.

**Case presentation:**

A 33-year-old female with a history of multiple sclerosis presented with new-onset neuropsychiatric symptoms. After substance-induced psychosis was ruled out, she was admitted to the medical ward for work up of psychosis secondary to multiple sclerosis. However, the consultation-liaison psychiatry service noted atypical symptoms which were concerning for autoimmune encephalitis. Admission to a psychiatric inpatient ward was deferred. Anti-NMDAR encephalitis was diagnosed with CSF analysis demonstrating lymphocytic pleocytosis and anti-NMDAR antibodies. In addition to first-line treatment of encephalitis with steroids, second-line immunotherapies were also implemented given the patient’s underlining demyelinating syndrome. The patient’s neurologic and psychiatric symptoms began to improve.

**Conclusions:**

There is literature to demonstrate a possible connection between anti-NMDAR encephalitis and demyelinating syndromes. As such, autoimmune encephalitis should be considered in patients with multiple sclerosis presenting with atypical symptoms. Determining the correct diagnosis is crucial to inform the appropriate treatment protocol, and to improve prognosis.

## Background

Anti-N-methyl-D-aspartate receptor (anti-NMDAR) encephalitis is an autoimmune disorder characterized by both neurologic and psychiatric symptoms. Neurologic symptoms can include amnesia, changes in consciousness, motor symptoms and seizures. Psychiatric symptoms commonly include anxiety, agitation, paranoia, delusions, hallucinations and even catatonia [1]. The disease predominantly affects younger individuals and females [2]. A large proportion of cases are associated with an identifiable tumor, most commonly ovarian teratoma [3]. Recommended management of anti-NMDAR encephalitis includes removal of the tumor (if present) and immunotherapy [1].

However, recent literature has also described an overlap of anti-NMDAR encephalitis and demyelinating syndromes [4]. Cases have been reported of anti-NMDAR encephalitis in patients with acute disseminated encephalomyelitis (ADEM) [5], optic neuritis [5–8], myelitis [6, 9] and multiple sclerosis [10–13]. This link is considered rare [14], however has important clinical implications as treatments and prognosis may differ [4].

Here, we describe a case of anti-NMDAR encephalitis in a female patient with a history of multiple sclerosis and no past psychiatric history, who initially presented with new-onset neuropsychiatric symptoms.

## Case presentation

Ms. W, a 33-year-old Caucasian female had a past medical history significant for multiple sclerosis, chronic low back pain and migraines. She initially presented to a rural hospital emergency department with confusion and was diagnosed with substance-induced psychosis and discharged with an Ativan prescription. She returned within days via police and emergency medical services and was admitted to the county’s psychiatric observation unit at our hospital for paranoia, thought disorganization, irritability, and impulsivity.

Communication with her primary care provider, family and neurologist indicated a past medical history of multiple sclerosis (diagnosed three years prior), for which she has not been under treatment. She had recently also experienced significant weight loss that year, and anxiety, paranoia and hyperactivity were noted by her mother within the past month. Speech abnormalities were also noted, as she would stutter or stammer when trying to express herself. Particular themes from the patient’s thought content included: having a tampon blocking her vagina, being pregnant, being an immigrant, and a general sense of deserving punishment. Later, her speech abnormalities evolved to reduced verbal output overall. History of substance abuse informed a provisional diagnosis of substance-induced psychosis as urine toxicology was positive for cannabinoids. After 3 days of no improvement in symptoms, treatment with oral low dose atypical anti-psychotics was initiated; however adherence was infrequent due to paranoia.

No resolution of symptoms after 6 days led to reconsideration of her diagnosis. Given her history of multiple sclerosis, magnetic resonance imaging (MRI) of her brain was obtained on day 7 of admission. MRI showed possible acute or subacute changes; particularly multifocal bilateral white matter lesions in the cerebral hemispheres and the signal characteristics of a few small lesions in the right posterior parietal and left occipital region that were suggestive of acute or sub-acute disease activity. However, this could not be confirmed as our radiologist did not have access to her previous scans for comparison. Internal medicine assessed patient and noted on exam diplopia on extreme gaze to the left side. Unfortunately, given the patient’s paranoia, she was unable to engage in cognitive screening tests such as a MOCA or MMSE. But staff observation of mental status fluctuation (waxing and waning of confusion) was considered. Extensive investigation for delirium was suggested and, on day 8 of admission, the patient was transferred to medicine to be under the care of the hospitalist.

Complete blood count, blood sugar, blood urea nitrogen, creatinine, electrolytes, troponin, liver function tests, thyroid stimulating hormone, rheumatoid factor, human immunodeficiency virus were all within normal limits or negative. Serum antinuclear antibodies (ANA), extractable nuclear antigen screen (ENA) and anti ribonucleoprotein (RNP) were positive. ANA positivity was characterized by a titre of 1:80 and a nucleolar pattern, raising suspicion for a possible systemic rheumatologic condition. However, all other serum antibody screening was negative. On day 10, Cerebrospinal fluid (CSF) analysis was pursued via lumbar puncture. The CSF protein level was within normal limits at 0.32 g/L and the CSF glucose level was within normal limits at 3.5 mmol/L. However, 39 total nucleated cells with 100% lymphocytes were noted. No organisms were noted on the gram stain. Herpes Simplex Virus (HSV) and Human Immunodeficiency Virus (HIV) tests were ordered. Acyclovir IV was started as the differential remained broad. HSV, HIV and hepatitis paneling returned negative. A neurotropic virus such as syphilis was also considered, but antibody screening was non-reactive. On the date of her lumbar puncture, the patient had reported recent crystal methamphetamine use. Consultation-liaison psychiatry services were consulted on day 13 to assess for the possibility of a substance induced-psychosis versus primary psychosis, and need for inpatient psychiatry.

It was noted on psychiatric assessment that Ms. W continued to present with anxiety, fear, and confusion. She had insight into her confusion and remarkable distress about her memory loss. She required constant reminders and redirection. Themes of paranoia remained. She denied perceptual disturbances. Her symptoms did not improve in the absence of substance use and the administration of antipsychotics. A diagnosis of possible autoimmune encephalopathy was considered and admission to a psychiatric inpatient setting was deferred. Antibody panels for neuronal surface antigens were still pending. Lorazepam 2 mg by mouth or intramuscular every 4 h as needed was ordered for agitation, favored over antipsychotics due to risk of catatonia. Steroids were recommended in the absence of an infectious cause. Neurology consultation was recommended for guidance on immunotherapy recommendations.

**Fig. 1:**
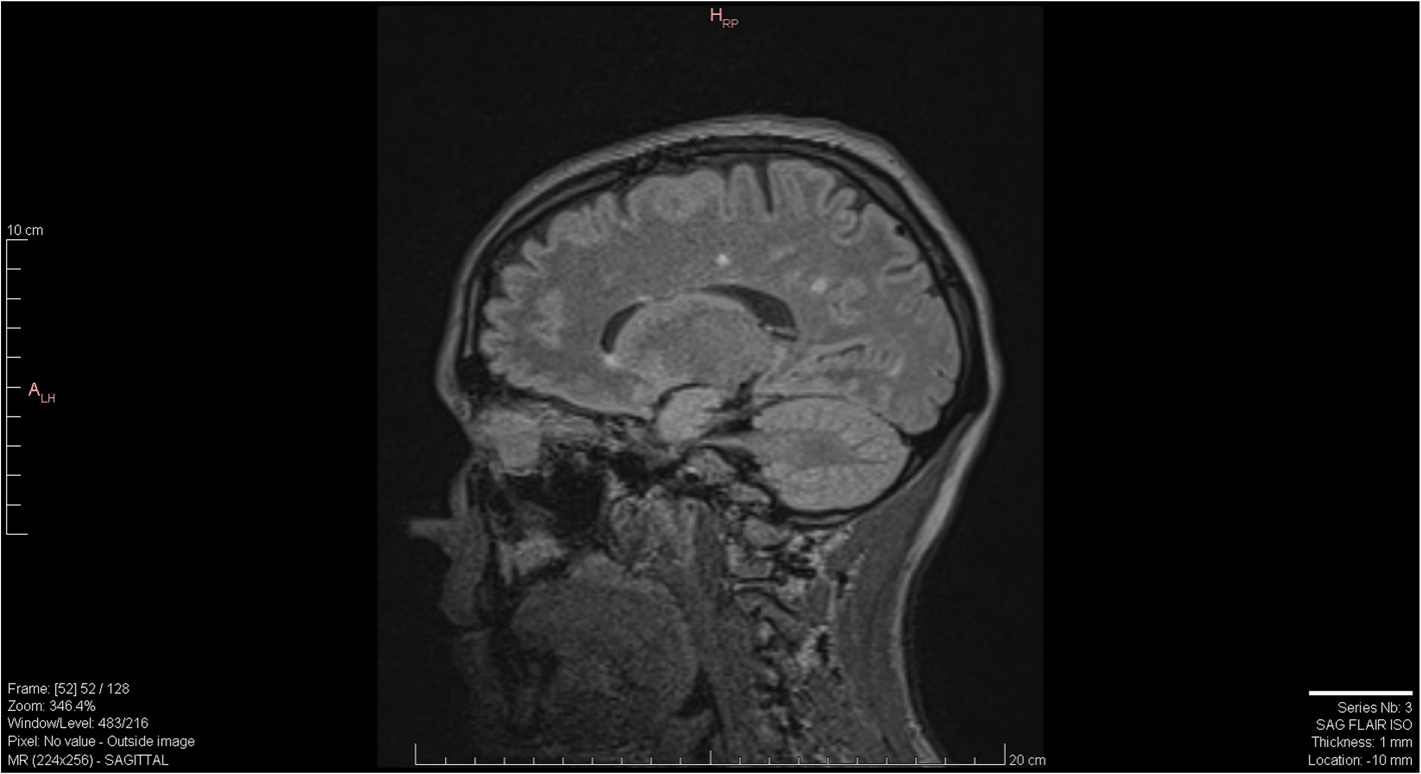
MRI SAG FLAIR 2020

**Fig. 2:**
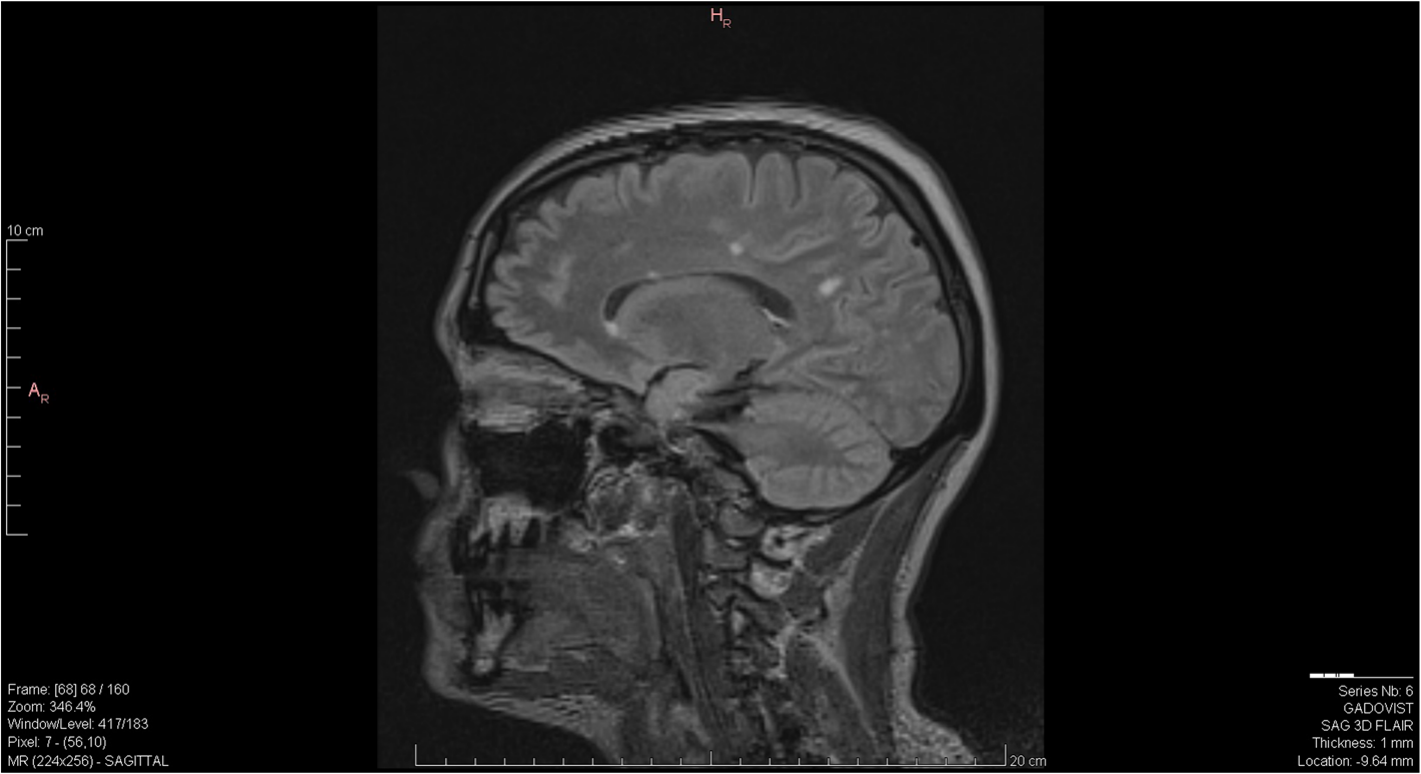
MRI SAG FLAIR 2019

**Fig. 3:**
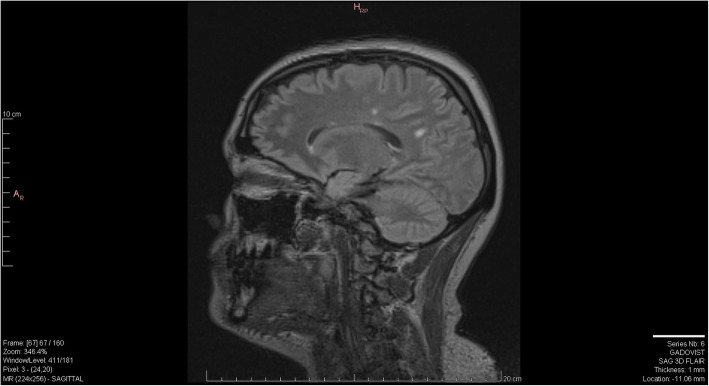
MRI SAG FLAIR 2018

Substance-induced psychosis versus primary psychosis remained the primary diagnostic consideration for her primary team for the following week and she was treated with risperidone, however her adherence to medication was sporadic. Her symptoms continued to fluctuate however she remained grossly thought disordered and anxious. Image records of previous MRI scans became available to us on day 18 from the outside facilities and radiology was able to compare those images to the MRI that the patient had received at our facility (Figs. 1, 2 and 3). An addendum was added post-comparison which indicated that other than one lesion in the splenium of the corpus callosum, white matter lesions were relatively unchanged from previous scans. Neurology consultation was obtained on day 19. Methylprednisolone 1 g intravenous (IV) every 24 h for three days was initiated for suspected autoimmune encephalitis now that progressive MS disease was not noted radiologically or clinically. A significant improvement in cognition was noted as she demonstrated improved arousal, attention, awareness, executive functioning and memory. Her paranoia of staff and overall fear also improved, however she remained somatically preoccupied with delusions of a damaged or blocked reproductive tract. On day 21, the results of her CSF antibody test (tested using EUROIMMUN cell-based assay in parallel with EUROIMMUN rat brain tissue IFA) returned positive for anti-NMDAR antibodies.

The diagnosis of Anti-NMDAR encephalitis was confirmed and she was transitioned to oral prednisone 50 mg daily. Intravenous immunoglobulin (IVIG) and plasmapheresis were also recommended. Computed tomography (CT) of chest and abdomen and ultrasound of pelvis ruled out malignancy. Ms. W received her first dose of IVIG and was subsequently transferred to the care of her primary neurologist outside of the county. There, she scored 27/30 on a Montreal cognitive assessment (MOCA), but some psychiatric symptoms remained. Prednisone was increased to 60 mg daily and IVIG was continued. At the time of writing this paper, she was scheduled for plasmapheresis and to initiate treatment with a monoclonal antibody to further treat her remaining symptoms. She was continued on an atypical antipsychotic.

## Discussion and conclusions

We present a rare case of anti-NMDAR encephalitis in a patient with a history of multiple sclerosis. An overlap between anti-NMDAR encephalitis and demyelinating syndromes has been reported. In 2014, a clinical and radiological analysis of a cohort of 691 patients by Graus and colleagues demonstrated that patients with anti-NMDAR encephalitis may develop concurrent or separate episodes of demyelinating disorders. Conversely, patients with demyelinating disorders who present with atypical symptoms may have anti-NMDAR encephalitis [4]. Our patient presented with neuropsychiatric symptoms that were atypical for her multiple sclerosis and raised suspicion for autoimmune encephalitis.

Her initial presentation was most notable for symptoms of anxiety, paranoia and disorganized thought. She was admitted to our psychiatric observation unit. This is common as the majority of anti-NMDAR encephalitis cases are initially seen by a psychiatrist [15]. Urine toxicology was positive for cannabinoids, which was suggestive of a possible substance-induced psychosis. However over subsequent days, her condition did not improve. The clinical observation of progressive symptomology in the absence of substance use made substance-induced psychosis unlikely. Level of consciousness was noted to fluctuate throughout the day. Collateral information indicated a history of multiple sclerosis and as such, concerns for psychosis secondary to multiple sclerosis were raised. MRI brain scan did reveal demyelinating lesions. However, once prior imaging records were obtained and compared to, these lesions were unchanged from previous scans. The lack of both radiologic and clinical signs of active disease ruled out a diagnosis of psychosis secondary to multiple sclerosis. Treatment history was also considered, as treatment with monoclonal antibodies has been described as a possible cause of encephalitis [16]. Our patient, however, had not been receiving such treatment. The primary team raised concern for substance-induced versus primary psychosis and requested admission to inpatient psychiatry, however our team deferred this transfer given the atypical features of this patient’s psychosis.

Our patient’s atypical features included disturbances of speech, rapid onset of cognitive deficits and a fluctuating course. These features in a person without prior psychiatric history are atypical for a primary psychiatric illness and raise suspicion for anti-NMDAR encephalitis [17]. Although MRI brain scan did not reveal new demyelinating lesions, it also did not demonstrate signs of encephalitis. But this is not uncommon as approximately half of anti-NMDAR encephalitis cases do not reveal MRI findings over the entire course of the disease [3]. As such, CSF antibody testing was pursued, which was positive for anti-NMDAR antibodies. This infers a mechanism of autoimmunity as antibodies to neuronal surface antigens have been implicated in synaptic dysfunction [18]. This has been proposed as a possible etiology for psychiatric symptoms, and would explain our patient’s phenotype and concern for psychiatric illness. This is further supported by our finding of prompt response to immunotherapy. The diagnosis of anti-NMDAR encephalitis was confirmed as lymphocytic pleocytosis and anti-NMDAR antibodies are the hallmark of this disease [1].

The co-occurrence of demyelinating disorders and anti-NMDAR encephalitis may be related to an activated autoimmune system [13]. Immunological studies often show independent but co-existing immune mechanisms underlying these disorders. The presence of aquaporin 4 (AQ4) and myelin-oligodendrocyte glycoprotein (MOG) antibodies have been associated with anti-NMDAR antibodies [4]. However, MOG antibody seropositivity has been described in cases of demyelination, absent of neuronal surface antibodies in the CS F[19]. Demyelinating features that are atypical for NMDAR encephalitis alone, such as unilateral sensorimotor deficits, may be explained by the presence of MOG antibodies [20]. As such, it is important to clarify a timeline for these patients and clinically correlate their laboratory workup with their current symptomology. Patients with encephalitis and antecedent history of demyelination will likely vary in clinical presentation from a patient with coexistence of these two disorders. The absence of clear sensorimotor deficits and radiological evidence of disease progression of multiple sclerosis guided us to conceptualize this case as an example of anti-NMDAR encephalitis in a patient with history of demyelination.

Immunotherapy is the mainstay of treatment for patients with anti-NMDAR encephalitis [1]. Although the first-line approach with steroids is similar for both anti-NMDAR encephalitis and demyelinating disorders, subsequent treatments can differ. Most patients with anti-NMDAR encephalitis do respond to first-line therapy, but patients with demyelination often require additional immunotherapies [4]. First-line immunotherapy includes steroids, IVIG and/or plasmapheresis. Second-line immunotherapies include monoclonal antibodies (such as rituximab) and/or cyclophosphamide [1]. Patients with both conditions may require an aggressive treatment regimen that includes first-line and second-line immunotherapies [6]. A common clinical challenge that exists in this patient population is the management of symptoms while awaiting antibody results. Antipsychotics are commonly prescribed to manage psychotic symptoms. However, NMDAR encephalitis patients can be resistant to treatment and require multiple psychotropics. Antipsychotics may be stopped due to concerns for developing NMS, for which this population is at high risk [17]. As such, benzodiazepines may be required. These considerations are important as a significant portion of patients with anti-NMDAR encephalitis remain severely disabled or die [15]. Patients with demyelinating episodes have been shown to require more intensive therapy and have more residual deficits [4]. This may necessitate longer-term use of psychotropics. This emphasizes the importance of early recognition and prompt treatment.

Although considered rare [14], the recognition that a demyelinating disorder and anti-NMDAR encephalitis may occur in the same patient has important diagnostic implications. Such patients are at great risk for misdiagnosis, as the diagnosis of anti-NMDAR encephalitis may not be considered and patients may not be adequately treated [4]. Therefore, prognosis can be affected as patients may be treated in psychiatric settings and exposed to unnecessary interventions. Or these patients may be treated only for their demyelinating disorder. As such, it is important to rule out anti-NMDAR encephalitis in patients with demyelinating disorders who demonstrate atypical symptoms of new onset psychosis [11].

## Data Availability

Not applicable.

## Abbreviations

ADEM: Acute disseminated encephalomyelitis
ANA: Antinuclear antibodies
AQ4: Aquaporin 4
CSF: Cerebrospinal fluid
HIV: Human immunodeficiency virus
HSV: Herpes simplex virus
IV: Intravenous
IVIG: Intravenous immune globulin
MOG: Myelin-oligodendrocyte glycoprotein
MS: Multiple sclerosis
MRI: Magnetic resonance imaging
NMDAR: N-methyl-D-aspartate receptor
NMS: Neuroleptic Malignant Syndrome
